# Long-Term Follow-Up of the Postoperative Macular Fold following the Vitreoretinal Surgery with Air Tamponade

**DOI:** 10.1155/2013/408351

**Published:** 2013-09-25

**Authors:** Ali Osman Saatci, Ozlem Barut Selver, Melih Parlak

**Affiliations:** ^1^Department of Ophthalmology, Dokuz Eylul University, 35320 Izmir, Turkey; ^2^Department of Ophthalmology, Buca Seyfi Demirsoy State Hospital, Izmir, Turkey; ^3^Department of Ophthalmology, Klinikum Konstanz, Luisenstrae 7, 78464 Konstanz, Germany

## Abstract

A 64-year-old male who had a macula-on superior bullous retinal detachment in OD underwent scleral buckling, 20-gauge-pars plana vitrectomy, internal drainage of subretinal fluid with perfluorocarbon fluid, 360° endolaser and perflourocarbon-fluid-air exchange surgery. Patient sat upright immediately after the surgery for the night. At the first postoperative morning although the retina was attached, there was a macular fold extending toward the temporal retinal periphery. Patient denied further surgery. During the follow-up, retinal fold gradually became less visible and it could be noticeable only by fundus autoflorescence imaging at the sixth postoperative year with a subtle epiretinal membrane formation on the optical coherence tomographic section.

## 1. Introduction

Retinal fold formation is a rare complication of retinal detachment (RD) surgery and has been described following intravitreal gas injection both with and without scleral buckling elements and with or without vitrectomy. It is thought to result from residual subretinal fluid being sequestered after the retinal break has been closed by intravitreal gas tamponade allowing subretinal fluid to accumulate in a gravity dependent position at the margin of attached and detached retina [1]. It may have a negative impact on visual outcome as histologic evidence demonstrates that the outer retina degeneration occurs as early as one week following the retinal fold formation and continues to worsen at four weeks [2].

We hereby report a case with macular fold that was developed following a successful scleral buckling surgery together with vitrectomy and fluid-air exchange for a bullous macula-on superior rhegmatogenous retinal detachment and describe its natural course over a six-year period.

## 2. Case Presentation

**Figure 1:**
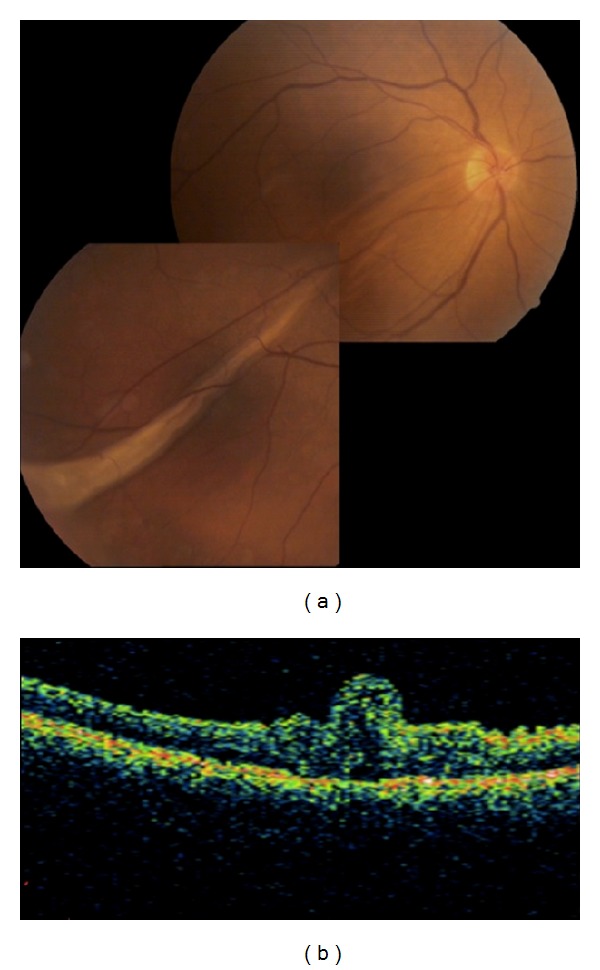
(a) Composite color fundus picture of the right eye taken two weeks after the surgery depicting the macular fold extending toward the temporal periphery of the retina. (b) OCT 3 image showing the macular fold.

A 64-year-old otherwise healthy man presented with a visual loss in his right eye of three days duration. Slit-lamp examination and intraocular pressure were normal with a clear lens in both eyes. On funduscopy, there was a macula-on superior bullous RD associated with four adjacent tears located at the superior quadrant in OD. The patient underwent encircling with an additional silicone tire extending from 10 to 2 hours in association with 20-gauge-pars plana vitrectomy, internal drainage of subretinal fluid (SRF) using perfluorocarbon liquid, 360° endolaser photocoagulation, and perflourocarbon-fluid-air exchange. Patient sat upright immediately after the surgery for the night. At the first postoperative morning, the retina was attached and the 2/3 of vitreous cavity was full of air. However, there was a significant macular fold extending toward the temporal retinal periphery. The patient was advised to take facedown position for the next five days. Two weeks later, macular fold remained almost nearly unchanged (Figures 1(a) and 1(b)).

**Figure 2:**
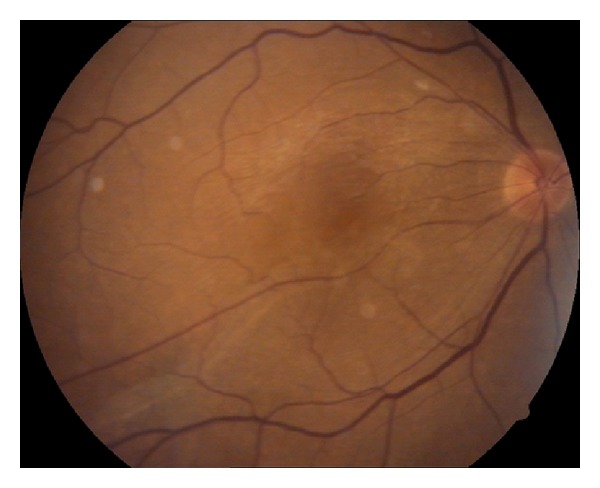
Right eye, color fundus picture at the ninth postoperative month showing the flattening of the fold.

Surgical option was discussed with the patient, and no further ocular surgery was carried out upon his denial. Visual acuity was 20/200 in his right eye. Nine months later, his visual acuity remained unchanged, and significant nuclear sclerosis was noted. However retinal fold looked less prominent (Figure 2).

**Figure 3:**
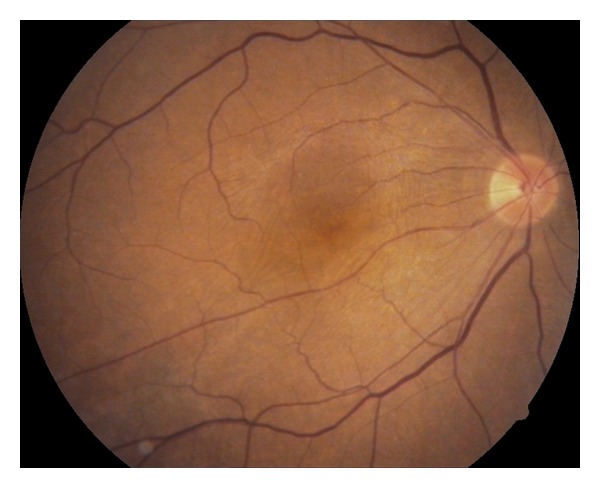
Right eye, color fundus picture taken at the third postoperative year.

He subsequently underwent an uncomplicated phacoemulsification and intraocular lens implantation at the 9th month post vitreoretinal surgery. At the third postoperative year his visual acuity was 20/100 and the fold looked almost gone (Figure 3).

**Figure 4:**
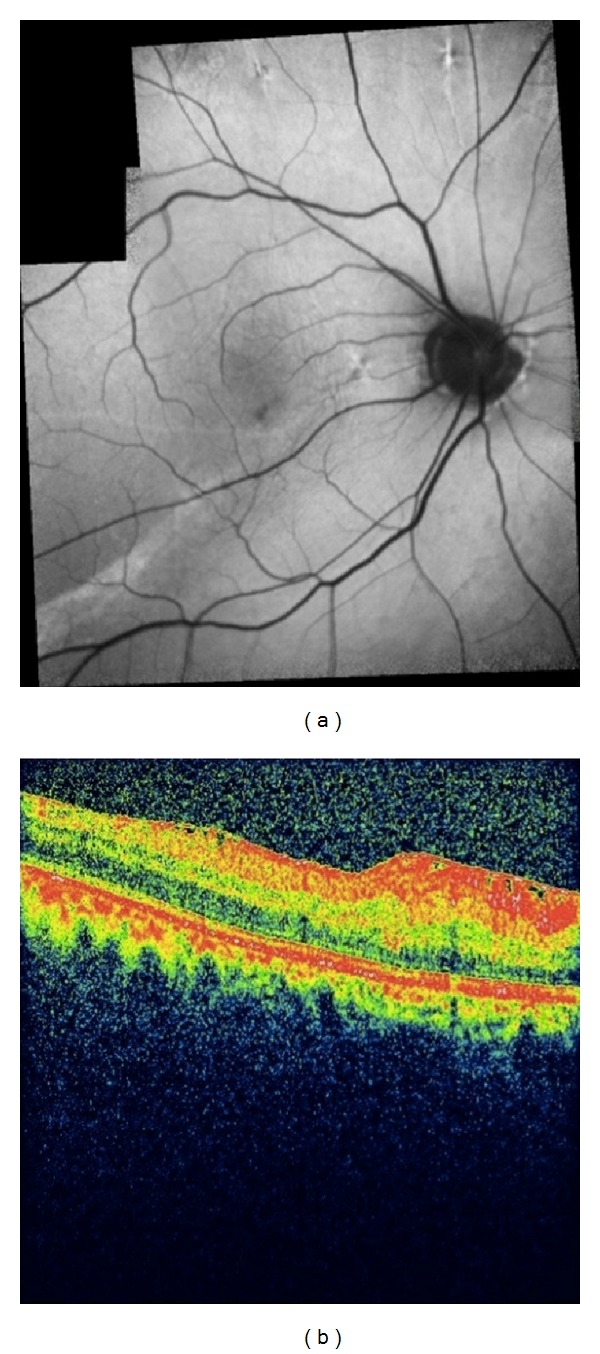
(a) Right eye, composite autoflorescent imaging showing the structural damage at the sixth postoperative year. (b) Spectral domain OCT demonstrating the slight epiretinal wrinkling in OD.

At the sixth postoperative year, the fold could be noticeable only by fundus autoflorescence imaging with slight epiretinal membrane formation on the optical coherence tomographic section. However his visual acuity remained the same (Figures 4(a) and 4(b)).

## 3. Discussion

There are very few reports about the macular fold formation after retinal detachment surgery [1]. One publication commented on the incidence in a consecutive series of 137 patients treated with retinal buckling surgery (encircling band and radial buckling) together with air-gas mixture injection, and the authors found a relatively high number of cases with macular folds (2.8%) [3].

Several risk factors were characterized: use of an intraocular gas tamponade, recent onset of retinal detachment and superior bullous detachment, large and circumferential buckle, external drainage of SRF, incomplete internal drainage of SRF in particular when performing primary vitrectomy, retinal detachment running through the fovea, and slippage of the retina following a retinal detachment with a giant tear [3–5].

Outcome varies among the cases. Complete regression of the retinal fold with full restoration of visual function, partial flattening with minimal or moderate improvement of visual function, or severe permanent structural damage are all possible consequences if the fold is left untreated [3, 5–8]. In most instances, surgeons elect to perform various types of surgical maneuvers to flatten the macular fold. In our case, the most likely cause was improper positioning taken immediately after the surgery. However, placement of silicone tire, insufficient subretinal fluid drainage, and bullous nature of the detachment might have all been contributed. Though macular fold was flattened without any surgical attempt and the color fundus picture almost looked normal, fundus autoflorescence definitely demonstrated permanent structural damage explaining the modest visual gain in our case.

In light of our case, we once again pointed out strongly the importance of immediate facedown position following any vitreoretinal surgery with air gas injection for rhegmatogenous retinal detachment to prevent macular fold formation in order not to endanger good visual outcome.
